# Research Advances on Pathways of Nickel-Induced Apoptosis

**DOI:** 10.3390/ijms17010010

**Published:** 2015-12-23

**Authors:** Hongrui Guo, Lian Chen, Hengmin Cui, Xi Peng, Jing Fang, Zhicai Zuo, Junliang Deng, Xun Wang, Bangyuan Wu

**Affiliations:** 1 Key Laboratory of Animal Diseases and Environmental Hazards of Sichuan Province, Sichuan Agricultural University, Ya’an 625014, China; guohonrui@163.com (H.G.); lianchen87@163.com (L.C.); pengxi197313@163.com (X.P.); fangjing4109@163.com (J.F.); zzcjl@126.com (Z.Z.); dengjl213@126.com (J.D.); wangxun99@163.com (X.W.); wubangyuan2008@163.com (B.W.); 2 College of Veterinary Medicine, Sichuan Agricultural University Ya’an, Ya’an 625014, China

**Keywords:** Ni, apoptosis, molecular mechanism, mitochondria, endoplasmic reticulum

## Abstract

High concentrations of nickel (Ni) are harmful to humans and animals. Ni targets a number of organs and produces multiple toxic effects. Apoptosis is important in Ni-induced toxicity of the kidneys, liver, nerves, and immune system. Apoptotic pathways mediated by reactive oxygen species (ROS), mitochondria, endoplasmic reticulum (ER), Fas, and c-Myc participate in Ni-induced cell apoptosis. However, the exact mechanism of apoptosis caused by Ni is still unclear. Understanding the mechanism of Ni-induced apoptosis may help in designing measures to prevent Ni toxicity.

## 1. Chemical Properties and Toxicity of Nickel

### 1.1. Chemical Properties of Ni

Nickel (Ni) is the 24th most abundant element in the earth’s crust [1,2]. It is the fifth heaviest element and is a part of group VIII B of the periodic table. In nature, Ni is found in combination with arsenic, antimony, and sulfur [3]. Elemental Ni is a silver-white solid metal, with high thermal conductivity, electrical conductivity, and melting point. Ni metal is highly stable [4] and is used for electroplating and protective coating. Ordinary Ni has an oxidation state of +2; higher oxidation states (such as Ni^+3^ and Ni^+4^) occur rarely in certain oxide systems [5]. Ni compounds are classified as soluble and insoluble [5,6]. Strong acid and organic acid Ni salts are soluble, whereas weak inorganic acid Ni salts are insoluble. Due to its chemical properties, gloss, and low price, Ni is used in jewelry, alloys, stainless steel, food processing industries, catalysts, and pigments [7,8,9]. Ni chloride, nitrate, sulphate, hydroxide, acetate, carbonate, and oxide are the most commercially important Ni compounds [10].

### 1.2. Biological Properties of Ni

Ni is essential for many microorganisms, plants, and animals [9,11,12]. Some studies have shown that very low levels of Ni are also essential for humans [13]. In rats, Ni deficiency reduces iron content in organs, haemoglobin, and hematocrit [14]. Ni has several biological functions, including activation of calcineurin; action and formation of cGMP [15]; transmission of genetic code (DNA, RNA) [16]; acting as a cofactor of albumin, proteins, and amino acids; transport of oxygen; stimulation of metabolism through interaction with iron in hemoglobin [17,18]; and formation of urease, carbon monoxide dehydrogenase, and methyl-*S*-coenzyme M reductase [15].

### 1.3. Ni Toxicity

The widespread use of Ni increases its concentration in biogeochemical cycles and increases human exposure by environmental contamination and occupational exposure [8]. Exposure to Ni commonly occurs by ingestion of contaminated water and food [9,19]. Workers in Ni-producing and processing industries are exposed by inhalation and, to a lesser extent, dermal contact [20]. People may also be exposed through contact with stainless steel, jewelry, and coins. Ni is toxic at high doses to both humans and animals [21]. Exposure to Ni can cause allergy, contact dermatitis, and toxicity of organ systems [22]. Li *et al.* [23] reported that NiCl_2_ increases the secretion of a pro-inflammatory cytokine, interleukin-1β (IL-1β), in bone marrow-derived macrophages and bone marrow dendritic cells. Ni may cause neurotoxicity, hepatotoxicity, nephrotoxicity, gene toxicity, reproductive toxicity, and increased risk of cancer [20,24,25,26,27,28,29,30,31,32]. Bones, kidneys, lungs, liver, and heart are the main organs of Ni accumulation [32,33,34].

Humans, especially Ni metallurgy workers, are exposed to Ni by inhalation and ingestion, with plants being the primary source of Ni for humans [34,35,36]. Ni may damage multiple organs and cause lung and nasal cancer [19,22,37]. Immediate and delayed hypersensitivity and allergic skin reactions are common adverse effects of Ni. Ni is both an allergen and a potential immunomodulatory and immunotoxic agent [38]. With the exception of metallic Ni, all Ni compounds have been classified as human carcinogens by the International Agency for Research on Cancer, based on studies on Ni workers and laboratory animals [39].

Numerous *in vitro*, *in vivo*, and epidemiological studies have documented the carcinogenic quality of Ni [40,41,42,43,44,45]. The LD50 of oral nickel acetate in rats and mice are 350 and 420 mg/kg, respectively [39]. Dietary NiCl_2_ at ≥300 mg/kg can cause reduced growth rate, and at ≥1100 mg/kg it can cause anemia or death in chickens [12]. Growth inhibition occurs at ≥700 mg/kg nickel sulfate (NiSO_4_) and nickel acetate in chicks [46]. Chicks fed a diet containing ≥250–300 mg/kg Ni can show inhibition of growth and decreased feed intake [47]. Oral NiCl_2_ decreases body and liver weight in mice [25]. Amudha *et al.* [48] have suggested that intraperitoneal NiCl_2_ causes significant kidney damage and reduced activities of enzymatic and non-enzymatic antioxidants in rats [48]. Dietary NiCl_2_ at ≥300 mg/kg damages the intestines and kidney, and decreases the immune function of the spleen, thymus, and bursa of Fabricius in chickens [49,50,51,52,53,54,55,56,57].

## 2. Biological Characteristics of Cell Apoptosis

**Figure 1:**
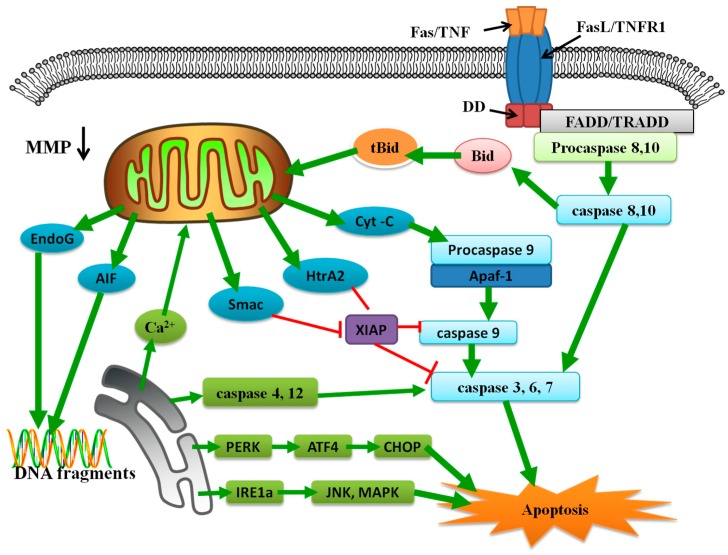
Summary of the extrinsic and intrinsic pathway in apoptosis. Extrinsic apoptosis: The combination of a ligand (FasL/TNFR1) and a death receptor (Fas/TNF) starts the extrinsic apoptosis pathway. This binding can attract the combination of FADD, TRADD and procaspase 8. Activated caspase 8 then activates downstream effector caspases or truncates the Bid. tBid can disrupt the mitochondria, and then induces mitochondria-mediated apoptosis. Intrinsic apoptosis: Mitochondria-mediated apoptosis pathway: The MMP disruption results in the release of cyt c, Smac, HtrA2, AIF and Endo G from mitochondrial intermembrane space to cytoplasm. Cytoplasmic cyt c promotes the aggregation of procaspase 9 and apaf1, and then activates caspase-9. Activated caspase-9 cleaves and activates caspase-3, 6, 7, which then induces apoptosis. Concurrently, Smac and HtrA2 inhibit XIAP expression for also contributing to the apoptosis. AIF and Endo G activate the caspase-independent mitochondria-mediated pathway. ER-mediate apoptosis pathway: Under prolonged ER stress, protein kinase RNA (PKR)-like ER kinase (PERK) pathway can induce apoptosis. Activated transcription factor 4 (ATF4) is an important transcription factor that accumulates via an un-conventional mechanism following PERK activation. ATF4 accumulation up-regulates the pro-apoptotic transcriptional factor, e.g., C/EBP homologous protein (CHOP), which induces apoptosis. The IRE1α pathway is also the important mechanism of ER stress. IRE1α mediates apoptosis through activation of c-Jun amino terminal kinase (JNK) and mitogen-activated protein kinase (MAPK) pathways. After the ER damage, acute translocation of Ca^2+^ from ER to mitochondria promotes Ca^2+^-mediated mitochondrial cell death. In addition, caspases-4 and -12 are also involved in ER stress-induced apoptosis.

Apoptosis is required for homeostasis of the cell population and defense during injury [58]. Failure to undergo apoptosis can cause several diseases such as cancer and autoimmune diseases, whereas excessive cell death is responsible for several neurodegenerative diseases [59]. So far, the main focus of research on the mechanism of apoptosis has been on the extrinsic and intrinsic apoptosis pathways (Figure 1) [60,61,62,63,64].

### 2.1. Bcl-2 Family Protein in Apoptosis

**Figure 2:**
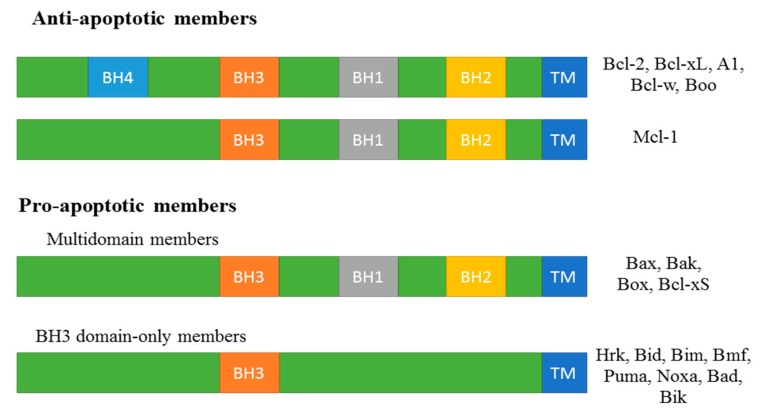
Bcl-2 Proteins. The Bcl-2 proteins can be subdivided into pro-survival and pro-apoptotic members, and have single or mutiple conserved functional Bcl-2 homology domains.

B-cell lymphoma-2 (Bcl-2) family proteins are involved in apoptosis [65], partly through the control and modulation of outer mitochondrial membrane integrity [66,67]. Based on their functions, Bcl-2 family proteins are classified into anti- and pro-apoptotic proteins (Figure 2). A loss of balance between anti- and pro-apoptotic proteins may cause either inhibition or promotion of apoptosis. Bcl-2 family members have single or multiple homology domains, such as Bcl-2 homology (BH1, -2, -3, and -4) that are important in the heterodimeric interaction among Bcl-2 family proteins [68]. Anti-apoptotic Bcl-2 family multi-domain proteins contain BH-(1-4) domains, such as Bcl-2, Bcl-2 homolog of ovary, Bcl-extra long (Bcl-xL), Bcl-w, and A1. Myeloid cell leukemia factor-1 (Mcl-1) is the only anti-apoptotic Bcl-2 protein with three BH domains: BH-1, -2, and -3 [69]. Based on the number of BH domains, pro-apoptotic Bcl-2 family proteins are classified into two subgroups. Bcl-2 antagonistic killer (Bak), Bcl-2 associated X protein (Bax), Bcl-2 related ovarian killer, and Bcl-extra short have multiple BH domains [68]. There are eight BH3 domain-only members: BH3 interacting domain death agonist (Bid), Bcl-2 interacting mediator of cell death (Bim), hara-kiri, p53-upregulated modulator of apoptosis (Puma), Bcl-2 modifying factor (Bmf), Bcl-2 antagonist of cell death (Bad), Noxa (named for “damage”), and Bcl-2 interacting killer [Bik, also known as natural born killer (Nbk)] [66,70]. In general, BH3 domain-only proteins suppress Bcl-2 anti-apoptotic proteins and induce apoptosis after lethal stress [71]. Bcl-2 maintains Bax and Bak in an inactive state. Furthermore, Bcl-2 titration away from Bax and Bak by BH3-only Bcl-2 family members allows them to oligomerize and form a channel through which cytochrome c (cyt c) can translocate to cytoplasm. Cyt c combines in a very specific stoichiometric fashion with apaf1 to form the apoptosome and activate caspase-9 [68,72].

Some studies have shown that p53 can regulate Bcl-2 proteins, but the specific mechanism is not clear [73]. The phosphoinositide-3-kinase/serine-threonine kinase (PI3K/Akt) pathway inhibits apoptosis via the modulation of Bcl-2 proteins [74,75,76]. This pathway increases anti-apoptotic proteins (Bcl-2 and Bcl-xL) and decreases pro-apoptotic proteins (Bad and Bax) [77,78,79,80].

### 2.2. Caspases in Apoptosis

**Figure 3:**
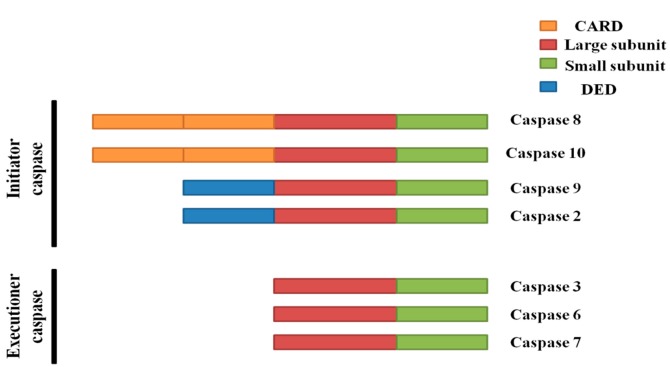
Caspase classification.

Caspases are cysteine proteases that are extremely important for intracellular apoptotic pathways [81,82,83]. After various intracellular and extracellular stimuli have occurred, caspases can be activated to execute apoptosis [84]. Pro-caspases are omnipresent in the cell, so apoptosis can occur rapidly without the need of transcription and translation. The removal of sick, damaged, and senescent cells provides a distinct survival advantage [85]. Caspases are divided into initiators and executioners, based on their physiological functions (Figure 3) [72,86,87,88,89].

Caspases-2, -8, -9 and -10 are initiators, as they are closely coupled to pro-apoptotic signals [90]. The initiator caspases exist in normal cells as inactive pro-caspase monomers and are activated by dimerization rather than cleavage. [91,92,93]. Dimerization promotes autocatalytic cleavage of caspase monomers into a large and a small subunit that results in dimer stabilization [94].

Caspases-3, -6, and -7 are executioners and exist as inactive pro-caspase dimers [95]. The initiator caspases activate executioner caspases by cleavage [94,96], which causes a conformational change that brings the two active sites of the executioner caspase dimer together and creates a functional mature protease [97]. Caspases-3 and -7 have identical cleavage sites. On being activated, an accelerated feedback loop of caspase activation occurs, ultimately causing cell death [97].

Murine caspase-12 and human caspase-4 belong to the interleukin-1-converting enzyme subfamily. Murine caspase-12 and human caspase-4 have 48% homology at the amino acid level and have structures similar to initiator caspases [98,99]. Caspase-12 and -4 are involved in endoplasmic reticulum (ER) stress-induced apoptosis [98,99,100].

### 2.3. Extrinsic Pathway in Apoptosis

The extrinsic apoptosis pathway is activated by the binding of extracellular death ligands to cell-surface death receptors [101]. The tumor necrosis factor (TNF) receptor gene super family is a member of the death receptors, which have a death domain (DD) that is important for apoptotic signal transduction [102,103]. FasL/FasR, TNF-α/TNFR1, Apo3L/DR3, Apo2L/DR4, and Apo2L/DR5 are the notable ligands and their corresponding death receptors [104,105,106].

FasL/FasR and TNF-α/TNFR1 are the characteristic models in the extrinsic apoptotic pathway. The extrinsic pathway is initiated by the binding of an extracellular death ligand to its cell surface transmembrane death receptor, causing oligomerization of the receptor [107]. This binding recruits the intracellular domain of the receptor (e.g., Fas associated death domain (FADD), TNF receptor associated death domain (TRADD)) and initiator caspases (e.g., procaspase-8 or -10) [108]. These complexes are regarded as death-inducing signaling complexes (DISC) [102,109,110,111]. The formation of these complexes promotes the oligomerization of procaspase-8 and -10 and auto-activation through self-cleavage [112].

Activated caspase-8 cleaves and activates downstream effector caspases such as caspase-1, -3, -6, and -7 [113]. As an important executioner of apoptosis, caspase-3 can activate many proteins, including the nuclear enzyme poly ADP-ribose polymerase (PARP) through proteolytic cleavage [114]. These downstream cleavage events are the morphological characteristics of apoptosis.

### 2.4. Intrinsic Pathway in Apoptosis

The intrinsic signaling pathways are known as mitochondria- and ER-initiated apoptosis [115,116].

#### 2.4.1. Mitochondria in the Intrinsic Pathway

The apoptotic stimuli cause changes in the Bcl-2 proteins. Bcl-2 titration away from Bax and Bak by BH3-only Bcl-2 family members allows them to oligomerize. This step opens the mitochondrial permeability transition pore (MPT) and disrupts the mitochondrial membrane potential (MMP), which translocates some pro-apoptotic proteins from the intermembrane space to the cytosol [117]. These pro-apoptotic proteins include cyt c, second mitochondrial activator of caspases/direct inhibitors of apoptosis binding protein with low pI (Smac/Diablo), the serine protease high-temperature-requirement protein A2/Omi (HtrA2/Omi), apoptosis inducing factor (AIF), and endonuclease G (Endo G) [61,118,119,120,121].

Apoptosis induced by cyt c, Smac/Diablo, and HtrA2/Omi requires the participation of caspases. Cyt c binds and activates apoptotic peptidase activating factor 1 (Apaf-1) and procaspase-9 to form an apoptosome [61]. The clustering of procaspase-9 in this apoptosome leads to caspase-9 activation. Caspase-9 activates downstream caspases, including caspase-3, -6, and -7, causing apoptosis. Inhibitors of apoptosis proteins (IAP) suppress apoptosis by directly inhibiting caspases [122,123]. Smac/DIABLO and HtrA2/Omi can promote apoptosis by inhibiting IAP proteins activity [124].

AIF and Endo G activate the caspase-independent mitochondrial pathway. After disruption of the mitochondrial membrane potential, AIF and Endo G translocate to the nucleus and cause DNA fragmentation and apoptosis [125,126,127].

#### 2.4.2. ER in the Intrinsic Pathway

Recent studies have shown that the ER also plays an important role in the intrinsic pathway [128,129]. The main function of ER is synthesis, folding, and translocation of proteins [130]. ER stress is activated by the disruption of ER function, which leads to the accumulation of misfolded and unfolded proteins in the ER lumen. ER stress induces an adaptive signal, Unfolded Protein Response (UPR), which restores ER homeostasis and protects the cell [116]. In the absence of this response, apoptosis occurs [116].

Under ER stress, protein kinase RNA (PKR)-like ER kinase (PERK) activates and phosphorylates eukaryotic translation initiation factor 2α (eIF2α), causing a translational arrest. However, under prolonged ER stress, it can promote apoptosis. Activated transcription factor 4 (ATF4) is an important transcription factor that accumulates via an unconventional mechanism following activation of the protein PERK [131]. Prolonged accumulation of ATF4 leads to apoptosis by a variety of mechanisms. ATF4 can upregulate the pro-apoptotic transcriptional factors, e.g., CHOP (C/EBP homologous protein, also called the growth arrest and DNA-damage-inducible 153, GADD153) [132]. CHOP promotes apoptosis through activation of downstream factors such as GADD34, a caspase-activating cell-surface death receptor of the tumor necrosis factor death receptor 5 (DR5), and endoplasmic reticulum oxidoreductase 1α (ERO1α) [133]. GADD34 inhibits the phosphorylation of elF2α [131]. This inhibition causes the accumulation of unfolded proteins in the ER lumen and translation of pro-apoptotic proteins. CHOP suppresses Bcl-2 transcription [134] and increases Bim expression [135], thereby promoting apoptosis. ERO1α activates the inositol triphosphate receptor (IP3R), which stimulates the translocation of excessive Ca^2+^ to the mitochondria and triggers the mitochondria-mediated apoptotic pathway [136]. ATF4 also regulates Noxa (a BH3-only protein) [137,138]. Noxa activates the effector proteins Bax and/or Bak [139] and induces mitochondria-mediated apoptosis.

The inositol-requiring enzyme/endonuclease 1α (IRE1α) pathway also plays an important part in ER stress. The IRE1α pathway has a pro-survival function, but it may cause apoptosis under prolonged ER stress [140]. IRE1α stimulates the activation of TNF receptor-associated factor 2 (TRAF2), which activates the apoptosis signal-regulating kinase 1/c-Jun amino terminal kinase (ASK1/JNK) cascade [141,142]. In addition, ER stress induces apoptosis through p38 mitogen-activated protein kinase (MAPK), which is activated by ASK1. p38 MAPK phosphorylates and activates CHOP and induces apoptosis [143]. Additionally, both p38 MAPK and JNK promote apoptosis through an increase in Bax [144].

The ER is the reservoir of Ca^2+^ in cells [145]. Ca^2+^ translocation from the ER to mitochondria triggers mitochondria-mediated apoptosis [146,147]. Excessive Ca^2+^ can disrupt the balance between Bcl-2 proteins and induce the translocation of cyt c [148].

In addition, some studies have suggested that caspase-12 is a specific marker of ER stress-induced apoptosis [128]. Mouse caspase-12 cleaves and activates caapase-9 and -3 and induces apoptosis [98,149]. However, human caspase-12 has no parallel function due to a frameshift disruption of its gene, resulting in a premature stop codon. In addition, human caspase-12 also contains amino acid substitutions in the caspase activity area [150]. Instead, human caspase-4 is specifically cleaved under ER stress, suggesting that it may be a functional ortholog of mouse caspase-12 in ER stress-induced apoptosis [99,151]. So far, little is known about how caspase-4 is activated in ER stress-triggered apoptosis.

## 3. Apoptosis Induced by Ni

Studies have demonstrated that Ni can increase apoptosis. Su *et al.* and Liu *et al.* [152,153] reported that NiSO_4_ induces DNA damage, apoptosis and oxidative damage in the liver and testes of mouse. Also, NiSO_4_ induces JNK-mediated oxidative damage and apoptosis in the liver of *Carassius auratus* [154]. NiCl_2_ has been proved to induce apoptosis in the liver of Kunming mice [153]. Our previous studies indicated that NiCl_2_ ≥ 300 mg/kg (dietary) can increase apoptosis percentages thymus, spleen, and cecal tonsil of broiler chickens [49,155,156].

*In vitro* research of Ni-induced apoptosis, Ni compounds can promote apoptosis in B cells [157], human T hybridoma cells [158], human hepatoma cells [159], keratinocytes [160], human airway epithelial (HEp-2) and human breast cancer (MCF-7) cells [161], human liver cells (HepG2) [162], and normal rat kidney cells [27], as well as human neutrophils and lymphocytes [163,164]. Shiao *et al.* [165] have reported that DNA fragmentation is detected in Chinese hamster ovary (CHO) cells treated with ≥160 μM Ni(II) and its intensity increases with increasing Ni(II) concentration.

## 4. Pathways or Mechanisms of Ni-Induced Cell Apoptosis

### 4.1. ROS-Mediated Apoptosis

Reactive oxygen species (ROS) acts as a crucial factor in the early stages of apoptosis. Mitochondria are both the source and target of ROS. When excess ROS is generated, it induces MMP depolarization and cyt c release, which triggers caspase activation [166,167,168,169]. The ROS generation can also induce DNA damage, which then promotes apoptosis [170].

Ni can induce oxidative stress *in vitro* and *in vivo* [171,172]. Ma *et al.* [173] demonstrated that Ni NWs induce apoptosis through ROS generation, and that ROS induces apoptosis through mitochondrial damage or activation of cell cycle checkpoints in HeLa cells. Nickel subsulfide (Ni_3_S_2_) induces ROS-mediated apoptosis in human bronchial epithelial cells (BEAS-2B) [174]. Ahamed [175] suggests that nickel nanoparticle (NiNPs) induces oxidative damage, which decreases glutathione (GSH), and induces ROS and lipid peroxidation (LPO) in human lung epithelial A549 cells.

Excess generation of ROS will result in oxidative stress that mediates apoptosis. The reduction of antioxidant defense is the main reason for ROS generation. Superoxide dismutase (SOD), catalase (CAT), glutathione reductase (GR), glutathione peroxidase (GSH-Px) and glutathione-*S*-transferase (GST) and GSH are the important antioxidant molecules. Some studies have reported that NiCl_2_ can decrease the antioxidative system in the *Tigriopus japonicas*, goldfish and broiler [48,50,53,155,156,176,177,178]. NiSO_4_ can decrease the GSH levels and activities of SOD and GSH-Px [154]. An amount of 13 uM Ni decreases CAT and GST activities in *M. galloprovincialis* (Lam) [179]. After exposure to NiNPs, ROS-induced apoptosis is observed in human skin epidermal cells [180]. However, Tyagi *et al.* [11] reported that renal SOD activity and GSH content are significantly increased after a high dose of NiCl_2_ has been used in rat.

Yet, Ni (II) at concentrations of 10–50 μM does not increase the intracellular ROS generation, but can activate the Nrf2 (NF-E2-related factor 2) signaling that is an antioxidant pathway [181]. The GSH depletion is due to the Ni-GSH complexes or Ni-mediated ROS formation [182].

### 4.2. The Extrinsic Pathway of Ni-Induced Apoptosis

At present, there are few studies on the extrinsic pathway of Ni-induced apoptosis. The results of Zhao *et al.* [183] suggest that metallic nickel particles can increase Fas, FADD, DR 3, and caspase-8 expression, but not DR6 and TNF-R2. Also, this study detects the formation of DISC. These data indicate that the extrinsic apoptotic pathway is involved in the apoptotic process caused by metallic nickel particles in JB6 cells. Bonin *et al.* [184] reported that higher expression levels of caspase-8 mRNA are detected in the Ni-exposed workers.

### 4.3. The Intrinsic Pathway of Ni-Induced Apoptosis

The mitochondria play a dominant role in the intrinsic apoptotic pathway [115,185]. Wang *et al.* [186] suggested that Nickel acetate induces MMP loss and apoptosis. Ni NWs-induced MMP disruption and apoptosis, and nickel ferrite nanoparticle-induced MMP loss are also found in HeLa cells [173] and in HepG2 and MCF-7 cells [187]. After the MMP loss, there are several apoptotic factor releases from the mitochondria. There is evidence that cyt c and caspases-9, -3 and -6 protein expression is increased after nickel acetate has been added in human proximal tubule cells [186]. Nickel nanoparticles and nickel fine particles can increase cyt c and AIF translocation from mitochondria to the cytoplasm [184]. AIF can induce apoptosis through caspase-independent pathway. However, cyt c can activate the caspase-dependent pathway. It has been demonstrated that fine nickel particles and nickel nanoparticles increase the protein expression and activation of caspase-3, -6, and -9 [183]. Additionally, NiSO_4_ increases caspase-3 activity in *Carassius auratus* liver [154]. Patel *et al.* [188] suggested that NiCl_2_ increases caspae-3, caspase-7 protein expression in human lung epithelial cells. Caspase-3 activity is obviously up-regulated with the increase in time and Ni NPs dose [175]. It has been also demonstrated that NiO nanoparticles (NiONPs) increases the numbers of apoptotic cells and the activaty of caspase-3 [189]. p53-defective human leukemic cells (U937) exposed to bis (*S*-citronellalthiosemicarbazonato) nickel(II) ([Ni(tcitr)2]) cause MMP disruption and caspase-3 activation [190]. Also, nickel nanoparticles (NiNPs) increases caspase-3 activity and apoptosis in A431 cells [180]. After exposure to nickel ferrite nanoparticles, the activation and gene expression of caspase-3 and caspase-9 are increased in HepG2 and MFC-7 cancer cells [187]. Dietary NiCl_2_ can increase caspase-3 expression in the thymus, spleen, and cecal tonsil in broiler chickens [49,155,156]. Abovementioned results show that the intrinsic signal pathway takes part in the apoptotic process induced by Ni and Ni compounds.

The Bcl-2 family of proteins is important in the modulation of the outer mitochondrial membrane integrity. In studies of Ni-induced apoptosis, Ni can alter the expression of Bcl-2 family proteins. Liu *et al.* [153] reported that NiCl_2_ can decrease the Bcl-2 protein expression and increase the Bax protein expression through suppression of PI3K/Akt pathway in the liver of Kunming mice. Ahamed *et al.* [187] suggest that nickel ferrite nanoparticles increase Bax mRNA expression through the p53 pathway, and decrease Bcl-2 mRNA expression. NiSO_4_ increases Bax protein expression and decreases Bcl-2 protein expression in *Carassius auratus* liver [154]. Reduction of Bcl-2 and Bcl-xL protein expression and enhancement of Bad, Bcl-Xs, Bax protein expression are observed after human proximal tubule cells and human bronchial epithelial cells have been cultured with nickel acetate and NiONPs [186,189]. Human BEAS-2B cells exposed to Ni_3_S_2_ show down-regulation of several antiapoptotic proteins (Bcl-2 and Bcl-xL) [174]. Dietary NiCl_2_ can increase Bax expression and Bax/Bcl-2 ratio and decrease Bcl-2 expression in the thymus, spleen, and cecal tonsil of broiler chickens [49,155,156]. However, the results of Zhao *et al.* [183] show that nickel nanoparticles and nickel fine particles can increase Bcl-2 expression and decrease Bax expression.

Up to now, there is only one report that nickel acetate can induce ER stress and increase CHOP protein expression in NRK52E cells and Hepa-1c1c7 cells [191].

### 4.4. Others

It has been shown that c-Myc amplifies the mitochondrial pathway and promotes and/or amplifies the death receptor pathway [192]. c-Myc can increase FasL expression, provoke combination of tBid and mitochondria, affect DISC components, inhibit Bcl-X expression and disrupt nuclear factor-κB (NF-κB) activation [193]. Besides, c-Myc activation provokes cyt c release [194]. c-Myc can selectively suppress Bcl-2 family proteins [193,195].

NiSO_4_ increases c-Myc protein and mRNA expression in non-tumorigenic Beas-2B and human keratinocyte HaCaT cells. It has been proved that the c-Myc is important in Ni-induced apoptosis through knockout and restoration technology. ERK/MEK (extracellular regulated protein kinases/mitogen-activated protein kinase) inhibitors (U0126 and PD98059) attenuate c-Myc expression. These results indicate that the ERK-dependent c-Myc pathway takes part in Ni-induced Beas-2B cell apoptosis [196].

## 5. Conclusions and Future Perspectives

**Figure 4:**
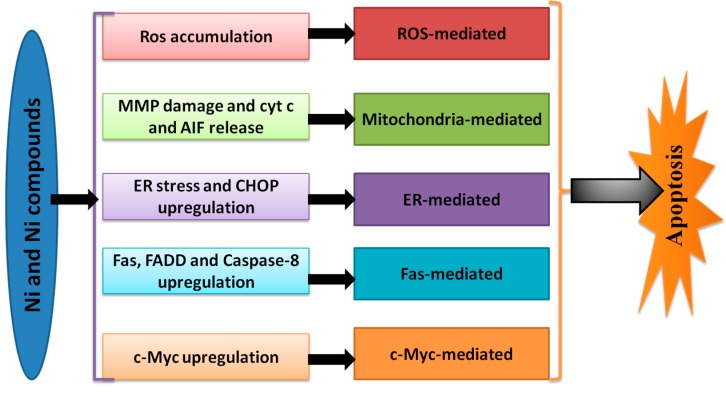
Pathways of Ni-induced apoptosis. The ROS-, mitochondria-, ER-, Fas- and c-Myc-mediated apoptotic pathway are all involved in Ni-induced cell apoptosis.

A number of studies have explored the molecular mechanism of Ni and Ni compounds-induced apoptosis. However, the precise mechanisms of Ni-induced apoptosis are inconclusive up to now. As shown in Figure 4, previous studies demonstrated that ROS-, mitochondria-, ER-, Fas- and c-Myc-mediated apoptotis are all involved in Ni-induced cell apoptosis. Most of these studies focus on the ROS- and mitochondria-mediated apoptosis. Only one study presents the ER-, Fas- and c-Myc-mediated apoptosis pathway. Therefore, more research should be conducted to explore whether ER-, Fas- and c-Myc-mediate apoptotic pathways are involved in Ni-induced apoptosis.

In addition to the conventional apoptotic proteins, another molecule has been found to regulate apoptosis. MicroRNAs (miRNAs) negatively regulate gene expression. Recent studies have reported that miRNAs participate in development, differentiation, and cell apoptosis [197,198,199].

Disorders of apoptosis may be important in the Ni or/and Ni compound toxicology, which includes neurotoxicity, hepatotoxicity, renaltoxicity, toxicity of the autoimmune system, and carcinogenicity. Revealing the mechanisms of Ni-induced apoptosis may contribute to the prevention of Ni toxicity.

## References

[1] Greenwood N.N., Earnshaw A., Earnshaw A. (1997). Chemistry of the Elements.

[2] Doll R. (1984). Nickel exposure: A human health hazard. IARC Sci. Publ..

[3] Gurley L.R., Tobey R.A., Valdez J.G., Halleck M.S., Barham S.S. (1983). Biological availability of nickel arsenides: Toxic effects of particulate Ni5As2. Sci. Total Environ..

[4] Rando D., Steglitz U., Morsdorf G., Kaltwasser H. (1990). Nickel availability and urease expression in Proteus mirabilis. Arch. Microbiol..

[5] Schaumlöffel D. (2012). Nickel species: Analysis and toxic effects. J. Trace Elem. Med. Biol..

[6] Munoz A., Costa M. (2012). Elucidating the mechanisms of nickel compound uptake: A review of particulate and nano-nickel endocytosis and toxicity. Toxicol. Appl. Pharmacol..

[7] Holbrook J., Minocha J., Laumann A. (2012). Body piercing: Complications and prevention of health risks. Am. J. Clin. Dermatol..

[8] Henderson R.G., Durando J., Oller A.R., Merkel D.J., Marone P.A., Bates H.K. (2012). Acute oral toxicity of nickel compounds. Regul. Toxicol. Pharm..

[9] Cempel M., Nikel G. (2006). Nickel: A review of its sources and environmental toxicology. Pol. J. Environ. Stud..

[10] Grandjean P. (1984). Human exposure to nickel. IARC Sci. Publ..

[11] Tyagi R., Rana P., Gupta M., Khan A.R., Bhatnagar D., Bhalla P.J., Chaturvedi S., Tripathi R.P., Khushu S. (2013). Differential biochemical response of rat kidney towards low and high doses of NiCl_2_ as revealed by NMR spectroscopy. J. Appl. Toxicol..

[12] Ling J., Leach R. (1979). Studies on nickel metabolism: Interaction with other mineral elements. Poultry Sci..

[13] Wen Z.T., Burne R.A. (2002). Functional genomics approach to identifying genes required for biofilm development by Streptococcus mutans. Appl. Environ. Microbiol..

[14] Kirchgessner M., Perth J., Schnegg A. (1980). Deficient nickel supply and the content of calcium, magnesium and phosphorus inthe bone of growing rats. Arch. Tierernahr..

[15] Poonkothai M.V.B.S. (2012). Nickel as an essential element and a toxicant. Int. J. Environ. Sci..

[16] Coogan T.P., Latta D.M., Imbra R.J., Costa M. (1989). Effect of nickel(II) on DNA-protein interactions. Biol. Trace Elem. Res..

[17] Schnegg A., Kirchgessner M. (1976). Absorption and metabolic efficiency of iron in nickel deficiency. Int. J. Vitam. Nutr. Res..

[18] Tallkvist J., Tjalve H. (1997). Effect of dietary iron-deficiency on the disposition of nickel in rats. Toxicol. Lett..

[19] Haber L., Erdreicht L., Diamond G., Maier A., Ratney R., Zhao Q., Dourson M. (2000). Hazard identification and dose response of inhaled nickel-soluble salts. Regul. Toxicol. Pharmacol..

[20] Oller A.R., Costa M., Oberdorster G. (1997). Carcinogenicity assessment of selected nickel compounds. Toxicol. Appl. Pharmacol..

[21] Wataha J.C., Lockwood P.E., Schedle A., Noda M., Bouillaguet S. (2002). Ag, Cu, Hg and Ni ions alter the metabolism of human monocytes during extended low-dose exposures. J. Oral Rehabil..

[22] Das K.K., Das S.N., Dhundasi S.A. (2008). Nickel, its adverse health effects & oxidative stress. Indian J. Med. Res..

[23] Li X., Zhong F. (2014). Nickel induces interleukin-1β secretion via the NLRP3-ASC-caspase-1 pathway. Inflammation.

[24] Xu S., He M., Zhong M., Li L., Lu Y., Zhang Y., Zhang L., Yu Z., Zhou Z. (2015). The neuroprotective effects of taurine against nickel by reducing oxidative stress and maintaining mitochondrial function in cortical neurons. Neurosci. Lett..

[25] Gathwan K.H., Al-Karkhi I.H.T., Jaffar Al-Mulla E.A. (2012). Hepatic toxicity of nickel chloride in mice. Res. Chem. Intermed..

[26] Scutariu M.D., Ciupilan C. (2007). Nickel and magnesium effects in the rat kidney, treated with acid retinoic. Comparative study. Rev. Med. Chir. Soc. Med. Nat. Iasi..

[27] Chen C.Y., Lin T.K., Chang Y.C., Wang Y.F., Shyu H.W., Lin K.H., Chou M.C. (2010). Nickel (II)-induced oxidative stress, apoptosis, G2/M arrest, and genotoxicity in normal rat kidney cells. J. Toxicol. Environ. Health A.

[28] Goodman J.E., Prueitt R.L., Dodge D.G., Thakali S. (2009). Carcinogenicity assessment of water-soluble nickel compounds. Crit. Rev. Toxicol..

[29] Forgacs Z., Massanyi P., Lukac N., Somosy Z. (2012). Reproductive toxicology of nickel—Review. J. Environ. Sci. Health A.

[30] Duman F., Ozturk F. (2010). Nickel accumulation and its effect on biomass, protein content and antioxidative enzymes in roots and leaves of watercress (*Nasturtium officinale* R. Br.). J. Environ. Sci..

[31] Kasprzak K.S., Sunderman F.W., Salnikow K. (2003). Nickel carcinogenesis. Mutat. Res..

[32] Spears J.W., Harvey R.W., Samsell L.J. (1986). Effects of dietary nickel and protein on growth, nitrogen metabolism and tissue concentrations of nickel, iron, zinc, manganese and copper in calves. J. Nutr..

[33] Das K.K., Dasgupta S. (2000). Effect of nickel on testicular nucleic acid concentrations of rats on protein restriction. Biol. Trace Elem. Res..

[34] Ragsdale S.W. (1998). Nickel biochemistry. Curr. Opin. Chem. Biol..

[35] Denkhausa E.S.K. (2002). Nickel essentiality, toxicity, and carcinogenicity. Crit. Rev. Oncol. Hemat..

[36] Krockova J.Z., Massanyi P., Sirotkin A.V., Pivko J., Makarevich A.V., Lukac N., Capcarova M., Toman R., Polakova Z. (2011). Nickel induced structural and functional alterations in mouse Leydig cells *in vitro*. J. Trace Elem. Med. Biol..

[37] Shi Z. (1994). Nickel carbonyl: Toxicity and human health. Sci. Total Environ..

[38] Das K.K., Buchner V. (2007). Effect of nickel exposure on peripheral tissues: Role of oxidative stress in toxicity and possible protection by ascorbic acid. Rev. Environ. Health.

[39] IARC (1990). IARC Monograph on the Evaluation of Carcinogenic Risks to Humans.

[40] Cavallo D., Ursini C., Setini A., Chianese C., Piegari P., Perniconi B., Iavicoli S. (2003). Evaluation of oxidative damage and inhibition of DNA repair in an *in vitro* study of nickel exposure. Toxicol. Vitro.

[41] Salnikow K., Zhitkovich A. (2008). Genetic and epigenetic mechanisms in metal carcinogenesis and cocarcinogenesis: Nickel, arsenic, and chromium. Chem. Res. Toxicol..

[42] Xu Z., Ren T., Xiao C., Li H., Wu T. (2011). Nickel promotes the invasive potential of human lung cancer cells via TLR4/MyD88 signaling. Toxicology.

[43] Beyersmann D., Hartwig A. (2008). Carcinogenic metal compounds: Recent insight into molecular and cellular mechanisms. Arch. Toxicol..

[44] Chakrabarti S.K., Bai C., Subramanian K.S. (2001). DNA-protein crosslinks induced by nickel compounds in isolated rat lymphocytes: Role of reactive oxygen species and specific amino acids. Toxicol. Appl. Pharmacol..

[45] Lei Y.X., Chen J.K., Wu Z.L. (2001). Detection of DNA strand breaks, DNA-protein crosslinks, and telomerase activity in nickel-transformed BALB/c-3T3 cells. Teratog. Carcinog. Mutagen..

[46] Weber C.W., Reid B.L. (1968). Nickel toxicity in growing chicks. J. Nutr..

[47] Szilagyi M., Szentmihalyi S., Anke M. Changes in Some of the Biochemical Parameters in Ni and Mo Deficient Animals (Goat, Sheep, Pig, Chicken, Rat). http://agris.fao.org/agris-search/search.do?recordID=HU8200908.

[48] Amudha K., Pari L. (2011). Beneficial role of naringin, a flavanoid on nickel induced nephrotoxicity in rats. Chem. Biol. Interact..

[49] Tang K., Guo H., Deng J., Cui H., Peng X., Fang J., Zuo Z., Wang X., Wu B., Li J. (2015). Inhibitive Effects of Nickel Chloride (NiCl_2_) on Thymocytes. Biol. Trace Elem. Res..

[50] Tang K., Li J., Yin S., Guo H., Deng J., Cui H. (2014). Effects of nickel chloride on histopathological lesions and oxidative damage in the thymus. Health.

[51] Huang J., Cui H., Peng X., Fang J., Zuo Z., Deng J., Wang X., Wu B. (2014). Effect of dietary nickel chloride on splenic immune function in broilers. Biol. Trace Elem. Res..

[52] Huang J., Cui H., Peng X., Fang J., Zuo Z., Deng J., Wang X., Wu B. (2014). Downregulation of TLR4 and 7 mRNA expression levels in broiler’s spleen caused by diets supplemented with nickel chloride. Biol. Trace Elem. Res..

[53] Guo H., Wu B., Cui H., Peng X., Fang J., Zuo Z., Deng J., Wang X., Deng J., Yin S. (2014). NiCl_2_-down-regulated antioxidant enzyme mRNA expression causes oxidative damage in the broiler’s kidney. Biol. Trace Elem. Res..

[54] Wu B., Cui H., Peng X., Fang J., Zuo Z., Deng J., Huang J. (2013). Dietary nickel chloride restrains the development of small intestine in broilers. Biol. Trace Elem. Res..

[55] Yin S., Cui H., Peng X., Fang J., Zuo Z., Deng J., Wang X., Wu B., Guo H. (2015). Toxic effect of NiCl_2_ on development of the bursa of Fabricius in broiler chickens. Oncotarget.

[56] Guo H., Deng H., Cui H., Peng X., Fang J., Zuo Z., Deng J., Wang X., Wu B., Chen K. (2015). Nickel chloride (NiCl_2_)-caused inflammatory responses via activation of NF-κB pathway and reduction of anti-inflammatory mediator expression in the kidney. Oncotarget.

[57] Guo H., Cui H., Peng X., Fang J., Zuo Z., Deng J., Wang X., Wu B., Chen K., Deng J. (2015). Dietary NiCl_2_ causes G2/M cell cycle arrest in the broiler’s kidney. Oncotarget.

[58] Hengartner M.O. (2000). The biochemistry of apoptosis. Nature.

[59] Lin M.T., Beal M.F. (2006). Mitochondrial dysfunction and oxidative stress in neurodegenerative diseases. Nature.

[60] Pulido M. (2003). Metal-induced apoptosis: Mechanisms. Mutat. Res..

[61] Jiang X., Wang X. (2004). Cytochrome C-mediated apoptosis. Annu. Rev. Biochem..

[62] Schulze-Osthoff K., Ferrari D., Los M., Wesselborg S., Peter M.E. (1998). Apoptosis signaling by death receptors. Eur. J. Biochem..

[63] Green D.R., Reed J.C. (1998). Mitochondria and apoptosis. Science.

[64] Elmore S. (2007). Apoptosis: A review of programmed cell death. Toxicol. Pathol..

[65] Hockenbery D.M., Oltvai Z.N., Yin X.M., Milliman C.L., Korsmeyer S.J. (1993). Bcl-2 functions in an antioxidant pathway to prevent apoptosis. Cell.

[66] Chao D.T., Korsmeyer S.J. (1998). Bcl-2 family: Regulators of cell death. Annu. Rev. Immunol..

[67] Besbes S., Mirshahi M., Pocard M., Billard C. (2015). New dimension in therapeutic targeting of Bcl-2 family proteins. Oncotarget.

[68] Martinou J.C., Youle R.J. (2011). Mitochondria in apoptosis: Bcl-2 family members and mitochondrial dynamics. Dev. Cell.

[69] Hetz C. (2010). Bcl-2 protein family. Essential regulators of cell death. Preface. Adv. Exp. Med. Biol..

[70] Lavik A.R., Zhong F., Chang M.J., Greenberg E., Choudhary Y., Smith M.R., McColl K.S., Pink J., Reu F.J., Matsuyama S. (2015). A synthetic peptide targeting the BH4 domain of Bcl-2 induces apoptosis in multiple myeloma and follicular lymphoma cells alone or in combination with agents targeting the BH3-binding pocket of Bcl-2. Oncotarget.

[71] Kelly P.N., White M.J., Goschnick M.W., Fairfax K.A., Tarlinton D.M., Kinkel S.A., Bouillet P., Adams J.M., Kile B.T., Strasser A. (2010). Individual and overlapping roles of BH3-only proteins Bim and Bad in apoptosis of lymphocytes and platelets and in suppression of thymic lymphoma development. Cell Death Differ..

[72] Ola M.S., Nawaz M., Ahsan H. (2011). Role of Bcl-2 family proteins and caspases in the regulation of apoptosis. Mol. Cell. Biochem..

[73] Schuler M., Green D.R. (2001). Mechanisms of p53-dependent apoptosis. Biochem. Soc. Trans..

[74] Osaki M., Oshimura M.A., Ito H. (2004). PI3K-Akt pathway: Its functions and alterations in human cancer. Apoptosis Int. J. Program. Cell Death.

[75] Porta C., Figlin R.A. (2009). Phosphatidylinositol-3-kinase/Akt signaling pathway and kidney cancer, and the therapeutic potential of phosphatidylinositol-3-kinase/Akt inhibitors. J. Urol..

[76] Chen J., Yang H., Wen J., Luo K., Liu Q., Huang Y., Zheng Y., Tan Z., Huang Q., Fu J. (2015). NHE9 induces chemoradiotherapy resistance in esophageal squamous cell carcinoma by upregulating the Src/Akt/β-catenin pathway and Bcl-2 expression. Oncotarget.

[77] Franke T.F., Hornik C.P., Segev L., Shostak G.A., Sugimoto C. (2003). PI3K/Akt and apoptosis: Size matters. Oncogene.

[78] Aziz M.H., Nihal M., Fu V.X., Jarrard D.F., Ahmad N. (2006). Resveratrol-caused apoptosis of human prostate carcinoma LNCaP cells is mediated via modulation of phosphatidylinositol 3′-kinase/Akt pathway and Bcl-2 family proteins. Mol. Cancer Ther..

[79] Hu L., Sun Y., Hu J. (2010). Catalpol inhibits apoptosis in hydrogen peroxide-induced endothelium by activating the PI3K/Akt signaling pathway and modulating expression of Bcl-2 and Bax. Eur. J. Pharmacol..

[80] Pan J.J., Chang Q.S., Wang X., Son Y.O., Liu J., Zhang Z., Bi Y.Y., Shi X. (2011). Activation of Akt/GSK3β and Akt/Bcl-2 signaling pathways in nickel-transformed BEAS-2B cells. Int. J. Oncol..

[81] Bratton S.B., Salvesen G.S. (2010). Regulation of the Apaf-1 caspase-9 apoptosome. J. Cell Sci..

[82] Martin S.J., Green D.R. (1995). Protease activation during apoptosis: Death by a thousand cuts?. Cell.

[83] Fan T.J., Han L.H., Cong R.S., Liang J. (2005). Caspase family proteases and apoptosis. Acta Biochim. Biophys. Sin..

[84] Lam M., Bhat M.B., Nunez G., Ma J., Distelhorst C.W. (1998). Regulation of Bcl-xL channel activity by calcium. J. Biol. Chem..

[85] Parrish A.B., Freel C.D., Kornbluth S. (2013). Cellular mechanisms controlling caspase activation and function. CSH Perspect. Biol..

[86] McIlwain D.R., Berger T., Mak T.W. (2013). Caspase functions in cell death and disease. CSH. Perspect. Biol..

[87] Brentnall M., Rodriguez-Menocal L., de Guevara R.L., Cepero E., Boise L.H. (2013). Caspase-9, caspase-3 and caspase-7 have distinct roles during intrinsic apoptosis. BMC Cell Biol..

[88] Xu G., Shi Y. (2007). Apoptosis signaling pathways and lymphocyte homeostasis. Cell Res..

[89] Salvesen G.S. (2002). Caspases: Opening the boxes and interpreting the arrows. Cell Death Differ..

[90] De Calignon A., Fox L.M., Pitstick R., Carlson G.A., Bacskai B.J., Spires-Jones T.L., Hyman B.T. (2010). Caspase activation precedes and leads to tangles. Nature.

[91] Chang D.W., Xing Z., Capacio V.L., Peter M.E., Yang X. (2003). Interdimer processing mechanism of procaspase-8 activation. EMBO J..

[92] Boatright K.M., Deis C., Denault J.B., Sutherlin D.P., Salvesen G.S. (2004). Activation of caspases-8 and -10 by FLIP_L_. Biochem. J..

[93] Muzio M., Stockwell B.R., Stennicke H.R., Salvesen G.S., Dixit V.M. (1998). An induced proximity model for caspase-8 activation. J. Biol. Chem..

[94] Shi Y. (2002). Mechanisms of caspase activation and inhibition during apoptosis. Mol. Cell.

[95] Tait S.W., Green D.R. (2010). Mitochondria and cell death: Outer membrane permeabilization and beyond. Nat. Rev. Mol. Cell Biol..

[96] Earnshaw W.C., Martins L.M., Kaufmann S.H. (1999). Mammalian caspases: Structure, activation, substrates, and functions during apoptosis. Annu. Rev. Biochem..

[97] Cohen G. (1997). Caspases: The executioners of apoptosis. Biochem. J..

[98] Nakagawa T., Zhu H., Morishima N., Li E., Xu J., Yankner B.A., Yuan J. (2000). Caspase-12 mediates endoplasmic-reticulum-specific apoptosis and cytotoxicity by amyloid-β. Nature.

[99] Hitomi J., Katayama T., Eguchi Y., Kudo T., Taniguchi M., Koyama Y., Manabe T., Yamagishi S., Bando Y., Imaizumi K. (2004). Involvement of caspase-4 in endoplasmic reticulum stress-induced apoptosis and Aβ-induced cell death. J. Cell Biol..

[100] Wang Z., Liu Z., Cao Z., Li L. (2012). Allicin induces apoptosis in EL-4 cells *in vitro* by activation of expression of caspase-3 and -12 and up-regulation of the ratio of Bax/Bcl-2. Nat. Prod. Res..

[101] Schmitz I., Kirchhoff S., Krammer P.H. (2000). Regulation of death receptor-mediated apoptosis pathways. Int. J. Biochem..

[102] Locksley R.M., Killeen N., Lenardo M.J. (2001). The TNF and TNF receptor superfamilies: Integrating mammalian biology. Cell.

[103] Ashkenazi A., Dixit V.M. (1998). Death receptors: Signaling and modulation. Science.

[104] Suliman A., Lam A., Datta R., Srivastava R.K. (2001). Intracellular mechanisms of TRAIL: Apoptosis through mitochondrial-dependent and -independent pathways. Oncogene.

[105] Krammer P.H. (1999). CD95(APO-1/Fas)-mediated apoptosis: Live and let die. Adv. Immunol..

[106] Rubio-Moscardo F., Blesa D., Mestre C., Siebert R., Balasas T., Benito A., Rosenwald A., Climent J., Martinez J.I., Schilhabel M. (2005). Characterization of 8p21.3 chromosomal deletions in B-cell lymphoma: TRAIL-R1 and TRAIL-R2 as candidate dosage-dependent tumor suppressor genes. Blood.

[107] Danial N.N., Korsmeyer S.J. (2004). Cell death: Critical control points. Cell.

[108] Marino G., Niso-Santano M., Baehrecke E.H., Kroemer G. (2014). Self-consumption: The interplay of autophagy and apoptosis. Nat. Rev. Mol. Cell Biol..

[109] Scott F.L., Stec B., Pop C., Dobaczewska M.K., Lee J.J., Monosov E., Robinson H., Salvesen G.S., Schwarzenbacher R., Riedl S.J. (2009). The Fas-FADD death domain complex structure unravels signalling by receptor clustering. Nature.

[110] Kichev A., Rousset C.I., Baburamani A.A., Levison S.W., Wood T.L., Gressens P., Thornton C., Hagberg H. (2014). Tumor necrosis factor-related apoptosis-inducing ligand (TRAIL) signaling and cell death in the immature central nervous system after hypoxia-ischemia and inflammation. J. Biol. Chem..

[111] Curtin J.F., Cotter T.G. (2003). Live and let die: Regulatory mechanisms in Fas-mediated apoptosis. Cell Signal..

[112] Dickens L.S., Powley I.R., Hughes M.A., MacFarlane M. (2012). The “complexities” of life and death: Death receptor signalling platforms. Exp. Cell. Res..

[113] Lavrik I.N., Krammer P.H. (2012). Regulation of CD95/Fas signaling at the DISC. Cell Death Differ..

[114] Nicholson D.W., Ali A., Thornberry N.A., Vaillancourt J.P., Ding C.K., Gallant M., Gareau Y., Griffin P.R., Labelle M., Lazebnik Y.A. (1995). Identification and inhibition of the ICE/CED-3 protease necessary for mammalian apoptosis. Nature.

[115] Mishra N.C., Kumar S. (2005). Apoptosis: A mitochondrial perspective on cell death. Indian J. Exp. Biol..

[116] Logue S.E., Cleary P., Saveljeva S., Samali A. (2013). New directions in ER stress-induced cell death. Apoptosis Int. J. Program. Cell Death.

[117] Vaux D.L. (2011). Apoptogenic factors released from mitochondria. Biochim. Biophys. Acta.

[118] Du C., Fang M., Li Y., Li L., Wang X. (2000). Smac, a mitochondrial protein that promotes cytochrome c-dependent caspase activation by eliminating IAP inhibition. Cell.

[119] Duckett C.S. (2005). IAP proteins: Sticking it to Smac. Biochem. J..

[120] Garrido C., Galluzzi L., Brunet M., Puig P.E., Didelot C., Kroemer G. (2006). Mechanisms of cytochrome c release from mitochondria. Cell Death Differ..

[121] Vande Walle L., Lamkanfi M., Vandenabeele P. (2008). The mitochondrial serine protease HtrA2/Omi: An overview. Cell Death Differ..

[122] Deveraux Q.L., Reed J.C. (1999). IAP family proteins—Suppressors of apoptosis. Genes Dev..

[123] Hasegawa T., Suzuki K., Sakamoto C., Ohta K., Nishiki S., Hino M., Tatsumi N., Kitagawa S. (2003). Expression of the inhibitor of apoptosis (IAP) family members in human neutrophils: Up-regulation of cIAP2 by granulocyte colony-stimulating factor and overexpression of cIAP2 in chronic neutrophilic leukemia. Blood.

[124] Wu G., Chai J., Suber T.L., Wu J.W., Du C., Wang X., Shi Y. (2000). Structural basis of IAP recognition by Smac/DIABLO. Nature.

[125] Norberg E., Orrenius S., Zhivotovsky B. (2010). Mitochondrial regulation of cell death: Processing of apoptosis-inducing factor (AIF). Biochem. Biophys Res. Commun..

[126] Buttner S., Eisenberg T., Carmona-Gutierrez D., Ruli D., Knauer H., Ruckenstuhl C., Sigrist C., Wissing S., Kollroser M., Frohlich K.U. (2007). Endonuclease G regulates budding yeast life and death. Mol. Cell.

[127] Van Loo G., Schotte P., van Gurp M., Demol H., Hoorelbeke B., Gevaert K., Rodriguez I., Ruiz-Carrillo A., Vandekerckhove J., Declercq W. (2001). Endonuclease G: A mitochondrial protein released in apoptosis and involved in caspase-independent DNA degradation. Cell Death Differ..

[128] Urra H., Dufey E., Lisbona F., Rojas-Rivera D., Hetz C. (2013). When ER stress reaches a dead end. Biochim. Biophys. Acta.

[129] Sano R., Reed J.C. (2013). ER stress-induced cell death mechanisms. Biochim. Biophys. Acta.

[130] Yoshida H. (2007). ER stress and diseases. FEBS J..

[131] Iwasaki N., Sugiyama Y., Miyazaki S., Nakagawa H., Nishimura K., Matsuo S. (2015). An ATF4-signal-modulating machine other than GADD34 acts in ATF4-to-CHOP signaling to block CHOP expression in ER-stress-related autophagy. J. Cell. Biochem..

[132] Ohoka N., Yoshii S., Hattori T., Onozaki K., Hayashi H. (2005). TRB3, a novel ER stress-inducible gene, is induced via ATF4-CHOP pathway and is involved in cell death. EMBO J..

[133] Verfaillie T., Rubio N., Garg A.D., Bultynck G., Rizzuto R., Decuypere J.P., Piette J., Linehan C., Gupta S., Samali A. (2012). PERK is required at the ER-mitochondrial contact sites to convey apoptosis after ROS-based ER stress. Cell Death Differ..

[134] Hetz C.A. (2007). ER stress signaling and the Bcl-2 family of proteins: From adaptation to irreversible cellular damage. Antioxid. Redox Signal..

[135] Huang H.L., Wu J.L., Chen M.H., Hong J.R. (2011). Aquatic birnavirus-induced ER stress-mediated death signaling contribute to downregulation of Bcl-2 family proteins in salmon embryo cells. PLoS ONE.

[136] Li G., Mongillo M., Chin K.T., Harding H., Ron D., Marks A.R., Tabas I. (2009). Role of ERO1-α-mediated stimulation of inositol 1,4,5-triphosphate receptor activity in endoplasmic reticulum stress-induced apoptosis. J. Cell Biol..

[137] Zhu H., Yang W., He L.J., Ding W.J., Zheng L., Liao S.D., Huang P., Lu W., He Q.J., Yang B. (2012). Upregulating Noxa by ER stress, celastrol exerts synergistic anti-cancer activity in combination with ABT-737 in human hepatocellular carcinoma cells. PLoS ONE.

[138] Wang Q., Mora-Jensen H., Weniger M.A., Perez-Galan P., Wolford C., Hai T., Ron D., Chen W., Trenkle W., Wiestner A. (2009). ERAD inhibitors integrate ER stress with an epigenetic mechanism to activate BH3-only protein NOXA in cancer cells. Proc. Natl. Acad. Sci. USA.

[139] Gautam S., Kirschnek S., Wiesmeier M., Vier J., Hacker G. (2013). Roscovitine-induced apoptosis in neutrophils and neutrophil progenitors is regulated by the Bcl-2-family members Bim, Puma, Noxa and Mcl-1. PLoS ONE.

[140] Han D., Lerner A.G., Vande Walle L., Upton J.P., Xu W., Hagen A., Backes B.J., Oakes S.A., Papa F.R. (2009). IRE1α kinase activation modes control alternate endoribonuclease outputs to determine divergent cell fates. Cell.

[141] Jurczak M.J., Lee A.H., Jornayvaz F.R., Lee H.Y., Birkenfeld A.L., Guigni B.A., Kahn M., Samuel V.T., Glimcher L.H., Shulman G.I. (2012). Dissociation of inositol-requiring enzyme (IRE1α)-mediated c-Jun N-terminal kinase activation from hepatic insulin resistance in conditional X-box-binding protein-1 (XBP1) knock-out mice. J. Biol. Chem..

[142] Urano F., Wang X., Bertolotti A., Zhang Y., Chung P., Harding H.P., Ron D. (2000). Coupling of stress in the ER to activation of JNK protein kinases by transmembrane protein kinase IRE1. Science.

[143] Furuhata M., Takada E., Noguchi T., Ichijo H., Mizuguchi J. (2009). Apoptosis signal-regulating kinase (ASK)-1 mediates apoptosis through activation of JNK1 following engagement of membrane immunoglobulin. Exp. Cell Res..

[144] Newton V.L., Ali S., Duddy G., Whitmarsh A.J., Gardiner N.J. (2014). Targeting apoptosis signalling kinase-1 (ASK-1) does not prevent the development of neuropathy in streptozotocin-induced diabetic mice. PLoS ONE.

[145] Michalak M., Robert Parker J.M., Opas M. (2002). Ca^2+^ signaling and calcium binding chaperones of the endoplasmic reticulum. Cell Calcium.

[146] Timmins J.M., Ozcan L., Seimon T.A., Li G., Malagelada C., Backs J., Backs T., Bassel-Duby R., Olson E.N., Anderson M.E. (2009). Calcium/calmodulin-dependent protein kinase II links ER stress with Fas and mitochondrial apoptosis pathways. J. Clin. Investig..

[147] Rizzuto R., Pinton P., Carrington W., Fay F.S., Fogarty K.E., Lifshitz L.M., Tuft R.A., Pozzan T. (1998). Close contacts with the endoplasmic reticulum as determinants of mitochondrial Ca^2+^ responses. Science.

[148] Bravo R., Vicencio J.M., Parra V., Troncoso R., Munoz J.P., Bui M., Quiroga C., Rodriguez A.E., Verdejo H.E., Ferreira J. (2011). Increased ER-mitochondrial coupling promotes mitochondrial respiration and bioenergetics during early phases of ER stress. J. Cell Sci..

[149] Nakagawa T., Yuan J. (2000). Cross-talk between two cysteine protease families: Activation of caspase-12 by calpain in apoptosis. J. Cell Biol..

[150] Fischer H., Koenig U., Eckhart L., Tschachler E. (2002). Human caspase 12 has acquired deleterious mutations. Biochem. Biophys. Res. Commun..

[151] Bian Z.M., Elner S.G., Elner V.M. (2009). Dual involvement of caspase-4 in inflammatory and ER stress-induced apoptotic responses in human retinal pigment epithelial cells. Investig. Ophthalmol. Vis. Sci..

[152] Su L., Deng Y., Zhang Y., Li C., Zhang R., Sun Y., Zhang K., Li J., Yao S. (2011). Protective effects of grape seed procyanidin extract against nickel sulfate-induced apoptosis and oxidative stress in rat testes. Toxicol. Mech. Methods.

[153] Liu C.M., Zheng G.H., Ming Q.L., Chao C., Sun J.M. (2013). Sesamin protects mouse liver against nickel-induced oxidative DNA damage and apoptosis by the PI3K-Akt pathway. J. Agric. Food Chem..

[154] Zheng G.H., Liu C.M., Sun J.M., Feng Z.J., Cheng C. (2014). Nickel-induced oxidative stress and apoptosis in Carassius auratus liver by JNK pathway. Aquat. Toxicol..

[155] Wu B., Cui H., Peng X., Fang J., Zuo Z., Deng J., Huang J. (2014). Dietary nickel chloride induces oxidative stress, apoptosis and alters Bax/Bcl-2 and caspase-3 mRNA expression in the cecal tonsil of broilers. Food Chem. Toxicol..

[156] Huang J., Cui H., Peng X., Fang J., Zuo Z., Deng J., Wu B. (2013). The association between splenocyte apoptosis and alterations of Bax, Bcl-2 and caspase-3 mRNA expression, and oxidative stress induced by dietary nickel chloride in broilers. Int. J. Environ. Res. Public Health.

[157] Nowak M., Kopp F., Roelofs-Haarhuis K., Wu X., Gleichmann E. (2006). Oral nickel tolerance: Fas ligand-expressing invariant NK T cells promote tolerance induction by eliciting apoptotic death of antigen-carrying, effete B cells. J. Immunol..

[158] Guan F., Zhang D., Wang X., Chen J. (2007). Nitric oxide and Bcl-2 mediated the apoptosis induced by nickel(II) in human T hybridoma cells. Toxicol. Appl. Pharmacol..

[159] Kang J., Zhang D., Chen J., Lin C., Liu Q. (2004). Involvement of histone hypoacetylation in Ni^2+^-induced Bcl-2 down-regulation and human hepatoma cell apoptosis. J. Biol. Inorg. Chem..

[160] Cavani A. (2005). Breaking tolerance to nickel. Toxicology.

[161] Siddiqui M.A., Ahamed M., Ahmad J., Majeed Khan M.A., Musarrat J., Al-Khedhairy A.A., Alrokayan S.A. (2012). Nickel oxide nanoparticles induce cytotoxicity, oxidative stress and apoptosis in cultured human cells that is abrogated by the dietary antioxidant curcumin. Food Chem. Toxicol..

[162] Ahamed M., Ali D., Alhadlaq H.A., Akhtar M.J. (2013). Nickel oxide nanoparticles exert cytotoxicity via oxidative stress and induce apoptotic response in human liver cells (HepG2). Chemosphere.

[163] Freitas M., Barcellos-de-Souza P., Barja-Fidalgo C., Fernandes E. (2013). Nickel induces apoptosis in human neutrophils. Biometals Int. J. Role Metal Ions Biol. Biochem. Med..

[164] Chen C.Y., Wang Y.F., Huang W.R., Huang Y.T. (2003). Nickel induces oxidative stress and genotoxicity in human lymphocytes. Toxicol. Appl. Pharmacol..

[165] Shiao Y.H., Lee S.H., Kasprzak K.S. (1998). Cell cycle arrest, apoptosis and p53 expression in nickel(II) acetate-treated Chinese hamster ovary cells. Carcinogenesis.

[166] Venditti P., Di Stefano L., Di Meo S. (2013). Mitochondrial metabolism of reactive oxygen species. Mitochondrion.

[167] Dallas L.J., Bean T.P., Turner A., Lyons B.P., Jha A.N. (2013). Oxidative DNA damage may not mediate Ni-induced genotoxicity in marine mussels: Assessment of genotoxic biomarkers and transcriptional responses of key stress genes. Mutat. Res..

[168] Mates J.M., Segura J.A., Alonso F.J., Marquez J. (2012). Oxidative stress in apoptosis and cancer: An update. Arch. Toxicol..

[169] Avery S.V. (2011). Molecular targets of oxidative stress. Biochem. J..

[170] Simon H.U., Haj-Yehia A., Levi-Schaffer F. (2000). Role of reactive oxygen species (ROS) in apoptosis induction. Apoptosis Int. J. Program. Cell Death.

[171] Jomova K., Valko M. (2011). Advances in metal-induced oxidative stress and human disease. Toxicology.

[172] Valko M., Morris H., Cronin M. (2005). Metals, toxicity and oxidative stress. Curr. Med. Chem..

[173] Ma C., Song M., Zhang Y., Yan M., Zhang M., Bi H. (2014). Nickel nanowires induce cell cycle arrest and apoptosis by generation of reactive oxygen species in HeLa cells. Toxicol. Rep..

[174] Pan J., Chang Q., Wang X., Son Y., Zhang Z., Chen G., Luo J., Bi Y., Chen F., Shi X. (2010). Reactive oxygen species-activated Akt/ASK1/p38 signaling pathway in nickel compound-induced apoptosis in BEAS 2B cells. Chem. Res. Toxicol..

[175] Ahamed M. (2011). Toxic response of nickel nanoparticles in human lung epithelial A549 cells. Toxicol. Vitro.

[176] Kubrak O.I., Husak V.V., Rovenko B.M., Poigner H., Mazepa M.A., Kriews M., Abele D., Lushchak V.I. (2012). Tissue specificity in nickel uptake and induction of oxidative stress in kidney and spleen of goldfish Carassius auratus, exposed to waterborne nickel. Aquat. Toxicol..

[177] Wu B., Cui H., Peng X., Fang J., Zuo Z., Deng J., Huang J. (2013). Dietary nickel chloride induces oxidative intestinal damage in broilers. Int. J. Environ. Res. Publ. Health.

[178] Wang M., Wang G. (2010). Oxidative damage effects in the copepod *Tigriopus japonicus* Mori experimentally exposed to nickel. Ecotoxicology.

[179] Attig H., Kamel N., Sforzini S., Dagnino A., Jamel J., Boussetta H., Viarengo A., Banni M. (2014). Effects of thermal stress and nickel exposure on biomarkers responses in *Mytilus galloprovincialis* (Lam). Mar. Environ. Res..

[180] Alarifi S., Ali D., Alakhtani S., Al Suhaibani E.S., Al-Qahtani A.A. (2014). Reactive oxygen species-mediated DNA damage and apoptosis in human skin epidermal cells after exposure to nickel nanoparticles. Biol. Trace Elem. Res..

[181] Lewis J.B., Messer R.L., McCloud V.V., Lockwood P.E., Hsu S.D., Wataha J.C. (2006). Ni(II) activates the Nrf2 signaling pathway in human monocytic cells. Biomaterials.

[182] Krezel A., Szczepanik W., Sokolowska M., Jezowska-Bojczuk M., Bal W. (2003). Correlations between complexation modes and redox activities of Ni(II)-GSH complexes. Chem. Res. Toxicol..

[183] Zhao J., Bowman L., Zhang X., Shi X., Jiang B., Castranova V., Ding M. (2009). Metallic nickel nano- and fine particles induce JB6 cell apoptosis through a caspase-8/AIF mediated cytochrome c-independent pathway. J. Nanobiotechnol..

[184] Bonin S., Larese F.F., Trevisan G., Avian A., Rui F., Stanta G., Bovenzi M. (2011). Gene expression changes in peripheral blood mononuclear cells in occupational exposure to nickel. Exp. Dermatol..

[185] Susin S.A., Zamzami N., Castedo M., Hirsch T., Marchetti P., Macho A., Daugas E., Geuskens M., Kroemer G. (1996). Bcl-2 inhibits the mitochondrial release of an apoptogenic protease. J. Exp. Med..

[186] Wang Y.F., Shyu H.W., Chang Y.C., Tseng W.C., Huang Y.L., Lin K.H., Chou M.C., Liu H.L., Chen C.Y. (2012). Nickel (II)-induced cytotoxicity and apoptosis in human proximal tubule cells through a ROS- and mitochondria-mediated pathway. Toxicol. Appl. Pharmacol..

[187] Ahamed M., Akhtar M.J., Alhadlaq H.A., Khan M.A., Alrokayan S.A. (2015). Comparative cytotoxic response of nickel ferrite nanoparticles in human liver HepG2 and breast MFC-7 cancer cells. Chemosphere.

[188] Patel E., Lynch C., Ruff V., Reynolds M. (2012). Co-exposure to nickel and cobalt chloride enhances cytotoxicity and oxidative stress in human lung epithelial cells. Toxicol. Appl. Pharmacol..

[189] Duan W.X., He M.D., Mao L., Qian F.H., Li Y.M., Pi H.F., Liu C., Chen C.H., Lu Y.H., Cao Z.W. (2015). NiO nanoparticles induce apoptosis through repressing SIRT1 in human bronchial epithelial cells. Toxicol. Appl. Pharmacol..

[190] Buschini A., Pinelli S., Pellacani C., Giordani F., Ferrari M.B., Bisceglie F., Giannetto M., Pelosi G., Tarasconi P. (2009). Synthesis, characterization and deepening in the comprehension of the biological action mechanisms of a new nickel complex with antiproliferative activity. J. Inorg. Biochem..

[191] Hiramatsu N., Kasai A., Du S., Takeda M., Hayakawa K., Okamura M., Yao J., Kitamura M. (2007). Rapid, transient induction of ER stress in the liver and kidney after acute exposure to heavy metal: Evidence from transgenic sensor mice. FEBS Lett..

[192] Hoffman B., Liebermann D.A. (2008). Apoptotic signaling by c-Myc. Oncogene.

[193] Nilsson J.A., Cleveland J.L. (2003). Myc pathways provoking cell suicide and cancer. Oncogene.

[194] Juin P., Hueber A.O., Littlewood T., Evan G. (1999). c-Myc-induced sensitization to apoptosis is mediated through cytochrome c release. Genes Dev..

[195] Eischen C.M., Woo D., Roussel M.F., Cleveland J.L. (2001). Apoptosis triggered by Myc-induced suppression of Bcl-X(L) or Bcl-2 is bypassed during lymphomagenesis. Mol. Cell. Biol..

[196] Li Q., Suen T.C., Sun H., Arita A., Costa M. (2009). Nickel compounds induce apoptosis in human bronchial epithelial Beas-2B cells by activation of c-Myc through ERK pathway. Toxicol. Appl. Pharmacol..

[197] Cimmino A., Calin G.A., Fabbri M., Iorio M.V., Ferracin M., Shimizu M., Wojcik S.E., Aqeilan R.I., Zupo S., Dono M. (2005). miR-15 and miR-16 induce apoptosis by targeting Bcl2. Proc. Natl. Acad. Sci. USA.

[198] Jovanovic M., Hengartner M.O. (2006). miRNAs and apoptosis: RNAs to die for. Oncogene.

[199] Lima R.T., Busacca S., Almeida G.M., Gaudino G., Fennell D.A., Vasconcelos M.H. (2011). MicroRNA regulation of core apoptosis pathways in cancer. Eur. J. Cancer.

